# Systemic Lupus Erythematosus Features in Elderly Patients: Case-Based Review

**DOI:** 10.3390/jcm14082558

**Published:** 2025-04-08

**Authors:** Alexandr Ceasovschih, Raluca-Elena Alexa, Victorița Șorodoc, Andreea Asaftei, Denisa Cristiana Stoian, Bianca Codrina Morărașu, Anastasia Balta, Cătălina Lionte, Alexandra Stoica, Oana Sîrbu, Mihai Constantin, Alexandra-Diana Diaconu, Cristina-Mihaela Lăcătușu, Elena-Daniela Grigorescu, Laurențiu Șorodoc

**Affiliations:** 1 Faculty of Medicine, ‘Grigore T. Popa’ University of Medicine and Pharmacy, 700115 Iasi, Romania; alexandr.ceasovschih@yahoo.com (A.C.);; 2 2nd Internal Medicine Clinic, ‘Sf. Spiridon’ Clinical Emergency Hospital, 700111 Iasi, Romania; 3 Unit of Diabetes, Nutrition, and Metabolic Diseases, ‘Sf. Spiridon’ Clinical Emergency Hospital, 700111 Iasi, Romania

**Keywords:** systemic lupus erythematosus, late onset, elderly

## Abstract

**Background:** Systemic lupus erythematosus (SLE) is a heterogeneous autoimmune disease predominantly affecting young individuals; however, its late-onset manifestation poses distinct clinical and diagnostic challenges. **Methods:** This report describes the case of a 93-year-old patient who presented in the Emergency Department with exertional dyspnea, lower limb edema, fatiguability, diffuse abdominal pain, predominantly in the hypogastric region, and loss of appetite. **Results:** Based on the clinical examination, laboratory tests, and imagistic investigations, we excluded the most common etiologies of edema (decompensated chronic heart failure, glomerular nephropathy/chronic kidney disease, decompensated vascular cirrhosis, hypothyroidism, and hypoproteinemia). Further diagnostic evaluation revealed elevated levels of anti-nuclear antibodies and anti-dsDNA antibodies, along with reduced complement levels, indicating active SLE as the underlying cause of the patient’s edema. During hospitalization, the patient received corticosteroid therapy and, after discharge, was referred to the Rheumatology Department for further treatment. **Conclusions:** In elderly patients, late-onset SLE exhibits distinct clinical manifestations compared to its early-onset counterpart, likely due to age-related alterations in immune system function.

## 1. Introduction

Systemic lupus erythematosus (SLE) is a complex autoimmune disease, associated with autoantibodies, that leads to immune reactions and a wide range of clinical manifestations [1]. Usually, SLE involves the integumentary, renal, musculoskeletal, and hematologic systems and is diagnosed in young women between 20 and 40 years old [2]. In contrast, late-onset SLE, defined as disease onset after the age of 50, presents with a distinct clinical phenotype, with reduced mucocutaneous and renal involvement but a higher incidence of serositis, including pleuritis and pericarditis [3,4]. Additionally, the male-to-female ratio in late-onset SLE is lower than in younger patients and has a prevalence of less than 20% in Caucasian populations [5]. The nonspecific presentation of late-onset SLE frequently leads to diagnostic delays or misdiagnosis, as clinical manifestations may be erroneously attributed to age-related physiological changes [6].

We report the case of a female patient in whom generalized edema was the initial manifestation of SLE.

## 2. Case Presentation

A 93-year-old female patient was admitted to the Internal Medicine Clinic with a constellation of polymorphic symptoms. The symptomatology had insidiously developed over two months prior hospitalization, initially manifesting as diffuse abdominal pain, predominantly in the hypogastric region, loss of appetite, and fatigue, followed by exertional dyspnea and lower limb edema.

The patient’s medical history included arterial hypertension and hyperlipidemia, for which, at the time of the admission, she was receiving treatment with a beta-blocker, an anti-platelet agent, a dihydropyridine calcium channel blocker, and a statin.

Upon clinical examination, the patient was afebrile but with a moderately altered general status. She exhibited dyspnea on moderate exertion, cutaneous pallor, and symmetrical bilateral lower limb pitting edema. Cardiac auscultation revealed rhythmic but faint cardiac sounds, with a heart rate of 55 beats per minute and a blood pressure value of 145/70 mmHg. Peripheral pulses were weak bilaterally, likely secondary to edema. Notably, there were no clinical signs indicative of chronic venous insufficiency. On examination of the respiratory system, percussion of the thorax revealed dullness, and vesicular murmur in the lower third of both lung fields was absent. Oxygen saturation (SpO2) was 95% on room air. Abdominal examination revealed diffuse tenderness upon deep palpation, particularly in the hypogastric region.

Based on the patient’s history and the clinical examination, we identified the presence of edema and suggestive elements for pleural and pericardial effusions. Considering the clinical findings, our differential diagnosis included chronic decompensated heart failure, glomerular nephropathy/chronic kidney disease, decompensated liver cirrhosis, hypothyroidism, and hypoproteinemia. A comprehensive diagnostic workup, including laboratory investigations, functional assessments, and imaging studies, was initiated to determine the underlying etiology of the patient’s edema and pleural effusions.

**Figure 1:**
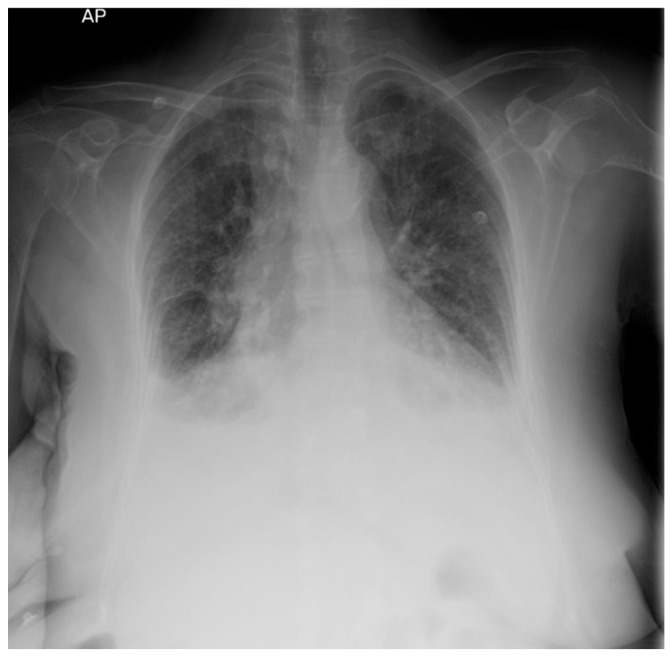
Chest X-ray: bilateral pleural effusion.

**Figure 2:**
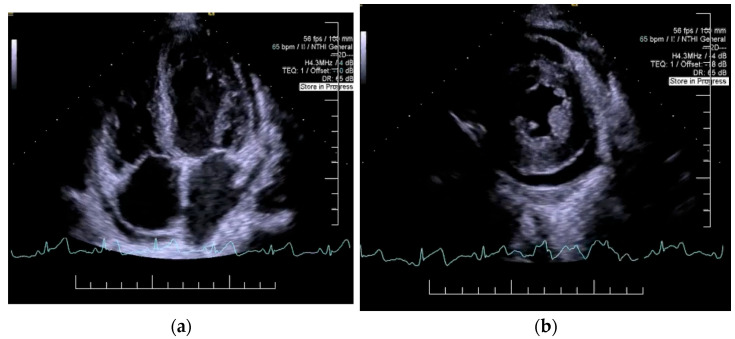
Echocardiography: left ventricular hypertrophy, impaired relaxation-type 1 diastolic dysfunction, (**a**) left ventricular ejection fraction of 50%, (**b**) circumferential pericardial effusion (9–11 mm).

Decompensated chronic heart failure was initially suspected due to the clinical presentation of exertional dyspnea, symmetric lower limb edema, and cardiopulmonary features suggestive of pleural and pericardial effusions. However, laboratory investigations revealed an NT-proBNP level of 418 pg/mL, well below the age-specific cut-off of 1800 pg/mL, reducing the likelihood of heart failure as the primary etiology. Electrocardiographic evaluation demonstrated sinus rhythm with a heart rate of 65 beats per minute and microvoltages in the unipolar limb leads. Chest X-ray identified bilateral pleural effusion and aortic atherosclerosis (Figure 1). H_2_FPEF score was 1, attributed solely to the patient’s advanced age. To achieve a more definitive diagnosis, transthoracic echocardiography was performed, which revealed a circumferential pericardial effusion (9–11 mm), left ventricular hypertrophy, impaired relaxation-type diastolic dysfunction, and a left ventricular ejection fraction (LVEF) of 50% (Figure 2). Aortic sclerosis was also noted. These findings, along with laboratory tests, established the diagnosis of chronic heart failure, but excluded decompensated heart failure as the underlying cause of the patient’s edema.

Renal ultrasound demonstrated symmetrically reduced kidney dimensions with otherwise normal morphology, and no evidence of urinary tract obstruction.

Decompensated vascular cirrhosis was also explored as a potential etiology, due to the patient’s fatigue and edema, but hepatic function tests were in normal ranges, without any biochemical elements of hepatocellular injury, cholestasis, hepatic insufficiency, or mesenchymal inflammation. Serological assays for hepatitis B and C were negative. Additionally, abdominal ultrasonography demonstrated normal hepatic and splenic morphology, with no signs of portal or splenic vein dilation and no ascites, further excluding cirrhosis as a contributing factor.

Hypothyroidism, another potential cause of generalized edema and serous effusions, was also considered. The presence of low-voltage electrocardiographic findings and bilateral pleural effusion prompted thyroid function testing, which yielded results within the normal range, thereby excluding hypothyroidism as an underlying cause of the patient’s clinical presentation.

The patient’s history of reduced oral intake, abdominal pain, and bowel movement disturbances raised concerns regarding protein-caloric malnutrition. However, there were no clinical signs of malnutrition, and laboratory investigations showed normal protein levels. Furthermore, following fluid redistribution and decongestion, the patient’s body mass index (BMI) remained within normal limits.

To further elucidate the etiology of the patient’s symptoms, particularly in the context of their advanced age and prior medical history, mesenteric ischemia and acute diverticulitis were considered as potential causes of abdominal pain. An abdominal X-ray was performed to exclude acute surgical emergencies such as intestinal obstruction or perforation. Additionally, laboratory analysis revealed mild normochromic, normocytic anemia, and a positive fecal occult blood test, suggestive of occult gastrointestinal hemorrhage.

Metabolic risk factors, including fasting plasma glucose, HbA_1c_, and lipid panel, were all within normal ranges.

Based on the presence of pleural and pericardial effusions, we conducted a series of investigations to establish the etiology of polyserositis.

Common differentials for polyserositis include malignancies (hematologic, non-Hodgkin lymphoma, breast, bronchopulmonary, gastric, pelvic, or colonic cancers), infections (Hepatitis B virus, Hepatitis C virus, Cytomegalovirus, Epstein–Barr virus, Coxsackie), autoimmune diseases (SLE, rheumatoid arthritis, adult-onset Still’s disease), and idiopathic cases. Considering the patient’s symptoms, including symmetric pitting edema, abdominal pain, and blood in stool, we suspected intra-abdominal venous or lymphatic obstruction, possibly due to a colonic neoplasm. Serological tumoral markers were elevated, with a notable increase in carbohydrate antigen 19-9 (CA 19-9), typically associated with pancreatic, colonic, or hepatobiliary neoplasms but also increased in autoimmune disease.

Imaging studies, including contrast-enhanced computed tomography (CT) of the chest, abdomen, and pelvis, were performed after the patient declined colonoscopy. Additionally, diagnostic thoracentesis was not feasible due to the minimal volume of pleural effusion. The CT scan confirmed bilateral pleural effusion, pericardial fluid, calcified atheromatous disease of the aorto-iliac vessels, and a 75% stenosis at the celiac trunk origin. Diverticula were observed in the sigmoid and descending colon, but without signs of complications.

Maintaining the suspicion of autoimmune polyserositis, we proceeded with testing for autoimmune causes. Rheumatoid factor was within normal limits, ruling out seroegative rheumatoid arthritis. Anti-nuclear antibodies (ANA) and anti-dsDNA antibodies were significantly elevated: over threefold the normal limit for ANA and over fivefold for anti-dsDNA antibodies, confirming the diagnosis of systemic lupus erythematosus (SLE). Complement levels (C3 and C4) were decreased, indicating active disease.

In the biological assessment, iron deficiency anemia was identified, with decreased serum levels of hemoglobin (11.5 g/dL), iron (17 μg/mL), and ferritin (38 ng/mL). Considering an autoimmune disease as the possible etiology of the edema, the anemia could have been hemolytic. However, the absence of reticulocytosis, along with normal serum levels of indirect bilirubin and lactate dehydrogenase, effectively ruled out hemolysis. Also, inflammatory markers were within normal ranges. Therefore, even if the patient presented with an active disease, the anemia was interpreted as the result of iron deficiency, probably due to the low intake of iron or gastrointestinal bleeding from the diverticula.

The primary therapeutic objectives during hospitalization were clinical stabilization through decongestive therapy, optimization of cardiovascular management, and initiation of targeted treatment for SLE. Loop diuretics (furosemide) were initially administered intravenously and subsequently transitioned to oral administration at a daily dose of 20 mg. SGLT-2 inhibitors (empagliflozin 10 mg daily) were administered to improve heart failure outcomes. Due to the presence of celiac trunk arterial stenosis and an increased cardiovascular risk profile, statin therapy (atorvastatin 40 mg daily) and antiplatelet therapy (acetylsalicylic acid 75 mg daily) were prescribed. Oral iron supplementation was initiated, with good gastrointestinal tolerance. Targeted immunosuppressive therapy for the SLE included intravenous methylprednisolone pulses (125 mg per day for three consecutive days), followed by oral prednisone (5 mg per day) as maintenance therapy, pending reassessment in the Rheumatology Clinic for treatment optimization. After treatment initiation, the patient showed signs of improvement: edema reduction, pain resolution, and absence of electrolyte imbalances. Imaging confirmed a slight reduction in pleural and pericardial effusions. Throughout hospitalization, renal and hepatic function parameters remained stable, and blood pressure and heart rate were within normal limits. Additionally, the patient demonstrated favorable gastrointestinal tolerance to corticosteroid therapy.

Upon discharge, the patient was advised to adhere to a low-sodium, fiber-rich diet with adequate hydration. A referral was made to the Rheumatology Department for treatment adjustment and continued management of SLE.

At the one-month follow-up, the patient exhibited marked clinical improvement, with complete resolution of lower limb edema and normalization of hemoglobin and serum iron levels. Imaging studies further confirmed a reduction in pericardial effusion.

The case highlights the multifaceted challenges of managing polyserositis in a 93-year-old patient with multiple associated comorbidities. After systematically evaluating the most common etiologies of systemic fluid retention in elderly patients, an autoimmune disease was considered a plausible underlying cause. Following the European Alliance of Associations for Rheumatology (EULAR)/American College of Rheumatology (ACR) classification criteria, the diagnosis of SLE was established. Given the potential renal implications of SLE, the patient’s renal function was assessed in accordance with the Kidney Disease: Improving Global Outcomes (KDIGO) 2024 Clinical Practice Guideline for the Management of Lupus Nephritis. At the time of evaluation, there was no evidence of SLE-induced glomerulopathy. Additionally, disease activity was quantified using the Systemic Lupus Erythematosus Disease Activity Index (SLEDAI), with the patient scoring 6 points, indicative of active disease.

Following confirmation of the diagnosis, renal function assessment, and disease activity evaluation, glucocorticosteroid therapy was initiated in accordance with the 2023 EULAR/ACR guidelines for SLE management to achieve rapid symptomatic relief. The uniqueness of this case is underscored by the late-onset presentation of SLE, its association with multiple age-related comorbidities, and the patient’s favorable response to immunosuppressive therapy. Notably, this patient represents the oldest documented case of SLE in the literature to date. The diagnostic and therapeutic approach posed a significant challenge in clinical practice due to the atypical symptomatology, the broad differential diagnosis of polyserositis in this age group, and the potential for adverse effects related to immunosuppressive therapy.

## 3. Discussions

### 3.1. Epidemiology

SLE is a complex disease that can affect multiple organ systems, primarily due to autoimmune abnormalities [7,8]. In elderly patients, SLE often presents differently compared to in younger individuals, with a typically lower overall disease activity. This phenomenon can lead to diagnostic challenges, as many of the symptoms are often attributed to the natural aging process, or senescence, rather than an underlying autoimmune condition [6,9]. Regarding the prevalence of SLE in the elderly, the available evidence is limited. Late-onset, defined as after 50 years old, SLE has an incidence of less than 20%, with most of the patients being women, but further epidemiological studies are required to establish prevalences in patients older than 80 years old [3,10].

For the purposes of our article, we selected the age threshold of 70 years, as this delineates the transition into the geriatric age group, a period marked by frailty and the need for specialized medical care. This age limit also aligns with definitions in the literature that distinguish between late- and very-late-onset SLE [11].

Systemic lupus erythematosus (SLE) is rare in the elderly, with distinct features such as equal gender distribution, insidious onset, and relatively benign course, leading to delayed diagnosis. Clinically, it primarily involves arthritis, serositis, myositis, pulmonary fibrosis, and Sjögren’s syndrome. Serologically, there is less hypocomplementemia and more frequent rheumatoid factor compared to in their younger counterparts. The disease is effectively managed with NSAIDs, hydroxychloroquine, low-to-moderate glucocorticoids, azathioprine, and methotrexate, without the need for mycophenolate mofetil, cyclophosphamide, or biologic agents. Despite a milder course, mortality is higher due to comorbidities, infections, and treatment complications [11,12].

In Table 1 are summarized all the clinical and biological characteristics of patients with late-onset SLE reported in the literature.

### 3.2. Pathophysiology

The primary mechanism of tissue damage in SLE is the development of auto-antibodies and the formation of circulating immune complexes, which are produced by inflammation brought on by dysregulation of immune system responses [2,41]. Inflammation and the adaptive immune system are triggered when processes such as complement activation or phagocytosis are disrupted [42]. In elderly patients, immunosenescence triggers mechanisms such as thymic involution, modifications in T-and B-cell functions, and decreased immune responses, leading to infections, chronic diseases, autoimmune diseases, or cancers [43].

#### 3.2.1. Apoptosis and Toll-like Receptors (TLRs)

Apoptosis is the physiological process by which phagocytes clear the cells programmed for death, preventing the accumulation of autoantigens [41]. Loss of self-tolerance appears when antigen-presenting cells (APCs) activate B and T cells and leads to the formation of autoantigens, stimulated by nucleoprotein complexes from late apoptotic cells that are not cleared by phagocytes [44,45]. Toll-like receptors (TLRs) serve as a bridge between the innate and adaptive immune systems. In SLE, TLR7 and TLR9 are particularly significant because they interact with DNA and RNA particles, which helps produce autoantibodies [46]. TLR activation, interferon regulatory factor 3 (IRF-3) activation, and nuclear factor kappa-light-chain-enhancer (NF-κB) activation all promote the production of type I interferon, which, in turn, stimulates T-cell proliferation and the generation of interferon-gamma (IFN-γ) [47,48].

#### 3.2.2. Innate Immune System

*Neutrophils*

In SLE, neutrophils exhibit impaired phagocytic activity, reduced production of reactive oxygen species (ROS), and increased formation of neutrophil extracellular traps (NETs). Abnormalities in ROS production contribute to the alteration of apoptosis and promote the generation of NETs, which subsequently trigger type I IFN production [41,49]. In elderly patients, despite elevated neutrophil counts, their functional capacity is compromised due to a diminished ability to form NETs and impaired chemotaxis [50,51].

*NET formation (NETosis)*

The immune response is influenced by NETs, which are structures that hold DNA, antigens, and other residues. The overproduction of NETs in SLE patients boosts the immune system and the production of autoantibodies [2,52,53].

*Natural killer cells (NK cells)*

Reduced NK cells seen in SLE are likely caused by elevated IFN-α levels or NK cell migration in target organs, most frequently the kidney. IFN-γ and tumor necrosis factor-alpha (TNF-α) are released when NK cells are activated, which promotes the development of immature NK cells and keeps the inflammation ongoing [41,54].

*Macrophages*

By generating pro-inflammatory cytokines like TNF-α, IFN-γ, IL-1β, and IL-12, as well as anti-inflammatory cytokines like IL-10 and IL-4, activated macrophages (Mf) trigger inflammatory and immune-modulatory responses [41]. The presence of Mf in the renal tissue leads to lupus nephritis [45].

*Basophils*

In addition to interacting with B cells by triggering their activation and the production of IgE, basophils have the ability to differentiate T cells into the Th2 type, which stimulates the production of inflammatory cytokines such as IL-4 and IL-6 [55].

*Dendritic cells*

By using cytokines and HLA-II molecules, dendritic cells (DCs) serve as antigen-presenting cells that link the innate and adaptive immune systems [41]. They can activate B cells and T cells, as well as produce IFN-α, which can result in the development of autoantibodies In patients with SLE, DCs modulate the immune response induced by immune complexes via TLRs [2,56].

#### 3.2.3. Adaptive Immune System

*T cells*

Follicular helper T cells (Tfh) contribute to B- and T-cell differentiation, autoantibodies, and the production of IL-21 and IL-4, cytokines that stimulate the germinal center B cells subgroup, which produces memory cells [56]. T-cell dysregulation and autoantibody production are caused by elevated levels of IL-12, IL-23, or transforming growth factor-beta (TGF-β), and levels of anti-dsDNA are directly connected with circulating Tfh [45,57].

Through the production of IL-17, Th-17 cells can stimulate inflammation by activating B cells and DCs, resulting in a pro-inflammatory state and elevated auto-antibodies [41,58].

Regulatory T cells (Tregs) exert a suppressive role in SLE; however, reduced levels of Tregs are associated with disease exacerbation. γδT cells can activate T cells and produce cytokines like IFN-γ, TNF-α, IL-10, IL-4, and IL-17, which lead to antibody formation [41,59,60].

*B cells*

Pro-inflammatory cytokines (TNF-α, IFN-γ, IL-2) activate BAFF, the key factor in the activation of B cells. Being a therapeutic target in SLE, high levels of BAFF are correlated with autoantibodies and SLE activity [45]. Tregs are activated by IL-5 and TGF-β, produced by regulatory B lymphocytes (Breg). Through TNF-α, IL-1, IL-6, IL-8, and inducible nitric oxide synthase (iNOS), anti-dsDNA autoantibodies cause and sustain inflammation [41].

#### 3.2.4. Complement System

The development of SLE is aided by the dysregulation of the complement system, which is a component of the innate immune system, and results in the improper removal of apoptotic debris. Through a variety of mechanisms, the complement system aids in the phagocytosis of antigen–antibody complexes. C1q antibodies are formed by mutations in the C1QA and C1QB genes, conformational alterations of C1q, or the presence of agents with molecular mimicry [61,62]. Lack of C4 causes B cells to proliferate and raises the number of autoantibodies as C4’s primary function is to suppress autoreactive B cells [63,64].

#### 3.2.5. Steroid Hormones

Steroid hormones are classified as sex hormones (androgen, estrogen, and progesterone) and corticosteroids (glucocorticoids and mineralocorticoids), all influencing the immune system [41].

The primary corticosteroid, cortisol, exerts anti-inflammatory effects through several mechanisms, including NF-κB activity inhibition, pro-inflammatory T-cell apoptosis, Treg cell survival, B-cell activity inhibition, proliferation, and antibody formation [65,66].

Aldosterone, the main mineralocorticoid, plays an inflammatory role through increasing NF-κB activity, DC activation, and B- and T-cell proliferation, leading to organ damage [67].

Sex hormones progesterone and androgen have a protective role against SLE, and estrogen contributes to SLE development by interfering with the immune system. Estrogen increases the production of IFN-α and contributes to the survival of autoreactive B cells and the production of IgG antibodies against ds-DNA. High levels of cortisol lead to Th2 cell activation and NETs formation. Progesterone reduces the production of IFN-α, contributes to the production of IgM antibodies, and decreases levels of IgG autoantibodies, and prevents formation of NETs. Androgens decrease the IgG autoantibody production and increase the TGF-β levels and NETosis [41,68,69].

#### 3.2.6. Physiopathology Particularities in Elderly Patients

Age-related immune system alterations in older patients include a pro-inflammatory state marked by a rise in cytokine production (TNF-α, IL-6, IL-1b, IL-18, CRP) and a decrease in cellular immunological responses [51,70]. These alterations could be explained by the inability of the innate immune system to recognize the pathogens and the inability of the adaptive immune system to produce antibodies, mechanisms that exacerbate and maintain the inadequate inflammatory response [43,71].

Alteration of the hematopoietic stem cells contributes to modifications in immune cell functions and composition, resulting in reduced T-cell activation and proliferation due to the decreased level of IL-7 production in the thymus [43].

Also, T cells are the most affected by aging. Loss of the CD28 receptor results in chronic inflammation through the production of IFN-γ, TNF-α, IL-8, granzyme B, and perforin [51,60]. In elderly patients, an elevated number of Tregs is observed, which may lead to altered memory cell response and the development of autoimmune diseases, potentially due to impaired Treg function [51]. Switching from the Th1 (cell-mediated) to the Th2 (humoral) response, along with the decreased production of naïve T cells, increased level of CD8+ T cells, and the loss of TCR sensitivity, enhances inflammation and the improper immune response [72].

Autoimmune response mediated by B cells is driven by the decreased production of naïve B cells and altered specificity of BCR, along with increased mature B cells, leading to a decline in humoral response [72,73,74].

In the elderly, myeloid DCs lose their antigen-presenting ability, fail to activate naïve T cells, and diminish the production of IL-12. Concurrently, plasmacytoid DCs lessen the production of type I and III IFN [72]. NF-kB and macrophage activation increase the IL-6 and TNF-α production, maintaining the low-grade inflammation and contributing to senescence [71]. Neutrophils’ phagocytosis ability is compromised even if their level is normal or increased, amplifying the inflammatory response [75,76].

Also, decreased estrogen levels induce lymphopenia due to the reactive oxygen species that induce DNA damage, predisposing women to autoimmune diseases [77] through epigenetic mechanisms and environmental factors described as predisposing ones. Estrogens generally enhance humoral autoimmunity and stimulate the T-cell response typical of lupus, leading to increased production of cytokines such as IL-4, IL-5, IL-10, and IL-13 [78,79]. The overlapping mechanisms lead not just to autoimmune component overexpression but also to excessive ROS formation, exacerbating SLE and affecting short- and long-term prognosis [80,81,82].

**Figure 3:**
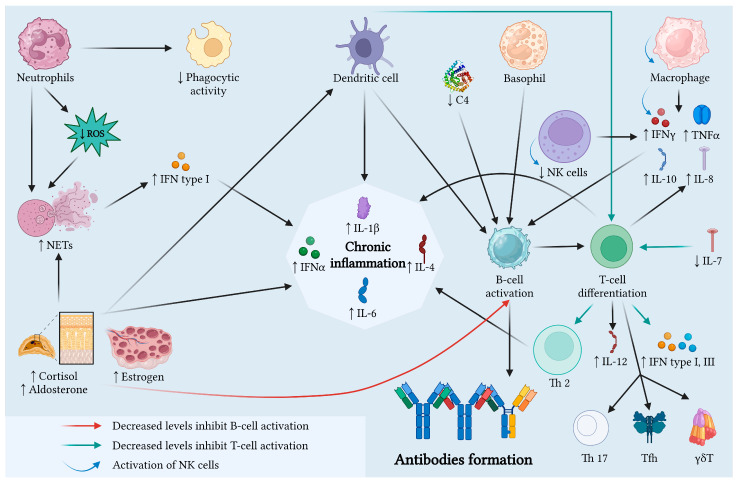
Physiopathological mechanisms in late-onset SLE. In the elderly, immunosenescence induces low-grade inflammation and maintains the alteration in the immune system. The pro-inflammatory state and the decrease in cellular immunological responses, due to the alteration in immune system components’ function, could explain the clinical manifestations, such as serositis or fibrosis. Legend: C4, complement component 4; IFN, interferon; IL, interleukin; NETs, neutrophil extracellular traps; NK, natural killer cells; ROS, reactive oxygen species; TNF, tumor necrosis factor. Created with BioRender.com.

Figure 3 summarizes the physiopathological mechanisms in late-onset SLE explained above.

### 3.3. Diagnosis

#### 3.3.1. Diagnosis Criteria

The diagnosis of SLE is based on a combination of clinical findings and laboratory tests organized through standardized criteria such as the ACR-1997, SLICC-2012, and EULAR/ACR-2019, which use a scoring system that considers the various manifestations of the disease. Currently, the EULAR/ACR-2019 criteria are preferred for diagnosing SLE as they were developed to increase the sensitivity of the SLICC-2012 criteria while maintaining the specificity of both the ACR-1997 and SLICC-2012 criteria. Having been validated in the adult population in 2020, these criteria have a sensitivity of 92% [83].

The diagnostic complexity of SLE is largely attributable to its marked heterogeneity. The absence of pathognomonic clinical features, coupled with its potential to affect any organ system, often contributes to delayed recognition. Diagnosis remains primarily clinical, supported by serological abnormalities. A high degree of clinical vigilance is warranted in patients exhibiting multisystemic involvement suggestive of SLE [83].

Over time, three main classification systems have been developed to characterize the clinical and immunological features of SLE: the ACR-1997, the SLICC-2012, and the more recent EULAR/ACR-2019 criteria. Although these classification criteria facilitate consistency in patient selection for clinical research, they are not intended for diagnostic purposes. Nevertheless, in routine clinical practice, they are frequently applied to guide the assessment of suspected cases [84].

Currently, the EULAR/ACR-2019 criteria are preferred for classifying SLE as they were developed to increase the sensitivity of the SLICC-2012 criteria while maintaining the specificity of both the ACR-1997 and SLICC-2012 criteria. Having been validated in the adult population in 2020, these criteria have a sensitivity of 92% [85].

#### 3.3.2. Characteristics of SLE in the Elderly Patient

Clinically, SLE in the elderly mainly manifests as serositis, myositis, pulmonary fibrosis, and Sjögren’s syndrome. As is summarized in Table 1, the most common manifestations in late-onset SLE include pleural effusion, lower limb edema, ascites, pericardial effusion, and arthralgia. Photosensitive lesions are less common in elderly patients, though not entirely absent. Serologically, elderly patients typically exhibit less hypocomplementemia and a higher prevalence of rheumatoid factor compared to younger individuals. The immunological alterations associated with aging may help explain the clinical differences observed between young and elderly patients [86].

In elderly patients, differential diagnosis for SLE should include other autoimmune diseases, such as rheumatoid arthritis, Raynaud syndrome, idiopathic thrombocytopenic purpura, polymyalgia rheumatica, or drug-induced lupus. In other autoimmune diseases, the diagnosis is based on clinical and biological modifications, but in lupus-like syndrome, in addition to the presence of clinical manifestations, patients could exhibit anti-nuclear antibodies, without any other immune serological alteration [87].

The management of elderly-onset SLE typically involves a conservative approach, with nonsteroidal anti-inflammatory drugs (NSAIDs), hydroxychloroquine, and low-to-moderate doses of glucocorticoids being the mainstay of treatment. Immunosuppressive agents such as azathioprine and methotrexate are commonly used, while mycophenolate mofetil, cyclophosphamide, and biologic agents are rarely required due to the generally milder course of the disease in this population. Despite a more indolent disease progression, elderly patients with SLE have higher mortality rates, primarily due to comorbidities, infections, and complications associated with treatment [11].

### 3.4. Management

**Figure 4:**
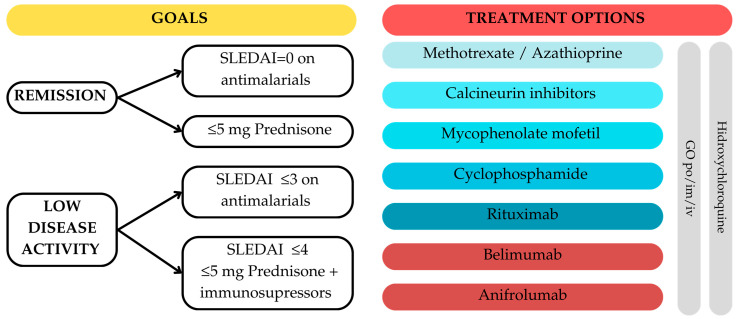
Treatment of SLE—EULAR 2023 recommendations for the management of SLE. GC = glucocorticosteroids; im = intramuscular; iv = intravenous; po = per os; SLEDAI = Systemic Lupus Erythematosus Disease Activity Index.

The management goal in patients with SLE is remission or low disease activity. Treatment options are listed in Figure 4.

In patients with SLE, regardless of the severity of the disease, hydroxychloroquine (HCQ) is the first-line treatment, even if the most important complication is retinopathy. The use of glucocorticosteroids is recommended for rapid symptom relief, but long-term use can lead to complications. There are multiple specimens for GC administrations, the main goal being to reduce the dosages to ≤5 mg Prednisone per day or stopping the administration [88].

When HCQ or GCs alone are insufficient to control disease activity, immunosuppressive drugs should be taken into consideration. The second line for mild forms of the condition is methotrexate (MTX); however, Azathioprine (AZA) should be considered in place of MTX if the patient is female and of childbearing age. Mycophenolate mofetil, cyclophosphamide, and rituximab are therapy choices for individuals with severe forms of the condition, while calcineurin inhibitors or mycophenolate mofetil may be administered to those with intermediate forms in addition to MTX and AZA. Immunosuppressive therapy with monoclonal antibodies such as B-lymphocyte stimulator-specific inhibitor (belimumab) and type 1 interferon receptor antibody (anifrolumab) are first-line treatment specimens in patients with severe disease but without renal involvement [88].

The management of late-onset SLE needs a multidisciplinary approach, considering the increased prevalence of comorbidities such as cardiovascular disease and infections, which can complicate treatment regimens [89,90]. The use of GCs and other immunesuppressive agents must be balanced against the risks associated with these therapies in older populations [91,92].

The age and general health of the patient must be carefully taken into account when treating late-onset SLE, especially when lupus nephritis is present. Research shows that individuals with late-onset lupus nephritis frequently receive less aggressive therapy than those with early-onset lupus, with immunosuppressive medications such as cyclo-phosphamide being significantly underutilized [93,94,95].

Clinically, late-onset SLE is characterized by milder symptoms, less renal involvement, and a lower total disease activity than early-onset cases [91,93]. Nevertheless, despite these variations, late-onset SLE is linked to a higher risk of mortality, which is frequently attributed to treatment-related problems and comorbidities rather than the illness itself [89].

### 3.5. Prognosis

Although elderly patients with late-onset SLE tend to have lower disease activity and less severe organ involvement, they are at higher risk for comorbidities that can exacerbate disease progression and lead to cumulative organ damage. Age at SLE onset is considered a significant risk factor for both mortality and morbidity due to accelerated atherosclerosis associated with chronic inflammation, long-term corticosteroid use, cardiovascular diseases, and osteoporosis [96].

Elderly patients with SLE often have pre-existing comorbid conditions such as hypertension, cardiovascular diseases, venous thrombosis, and pulmonary embolism, which can contribute to cumulative organ damage before the diagnosis of SLE [97]. These conditions, usually present at the time of SLE onset, can worsen the patient’s overall health and lead to further complications.

## 4. Conclusions

SLE is a heterogeneous autoimmune disorder that typically manifests between the second and fourth decades of life, predominantly in females. Late-onset SLE, defined as disease onset after the age of 50, can present diagnostic challenges due to the nonspecific nature of its manifestations, which may overlap with other chronic conditions commonly observed in elderly patients.

As previously discussed, patients with late-onset SLE often present with edema (including peripheral edema, pleural or pericardial effusion, and ascites), with less frequent involvement of joints or the characteristic malar rash. In these cases, when clinical symptoms cannot be attributed to other conditions, primary care physicians should consider an autoimmune etiology, and referral to a Rheumatology or Internal Medicine Clinic is warranted for further evaluation.

While the management guidelines for SLE do not specifically address the nuances of treatment in the elderly due to a lack of dedicated studies, it is crucial to exercise caution in these patients. Special attention should be paid to dose adjustments and close monitoring for adverse effects should be performed, particularly given the increased susceptibility to side effects in older populations.

The degree of cumulative organ damage is greater in patients with late-onset SLE, which is associated with a more complex risk profile due to advanced age. Aggressive prevention strategies are recommended for late-onset SLE patients to reduce the risk of cardiovascular diseases and osteoporosis. Patients such as those presented should benefit from a proactive treatment strategy and careful monitoring to prevent severe complications and improve their long-term quality of life.

## Figures and Tables

**Table 1:** Clinical and biological characteristics of patients with late-onset SLE.

Author, Year Number of Cases [Reference]	Age, Gender	Clinical Manifestation	Laboratory Test Abnormalities	Observations
So et al., 2019 3 cases [3]	75 y/o ♂	bilateral pleural effusion	positive tests for anti-dsDNA Ab hypocomplementemia pancytopenia, hematuria	In elderly patients with cardiovascular history, bilateral pleural effusion can mimic congestive heart failure. Thoracocentesis is essential for evaluating diuretic-resistant bilateral pleural effusion in elderly patients.
	80 y/o ♀	bilateral pleural effusion	positive tests for ANA hypocomplementemia leukopenia, proteinuria	
	83 y/o ♂	bilateral pleural effusion	positive tests for ANA, anti-dsDNA Ab, elevated CRP levels	
Ikushima et al., 2018 1 case [4]	85 y/o ♀	fatigue, bilateral lower extremity edema, arthritis, pleural effusion, pericardial effusion	positive tests for ANA, anti-dsDNA Ab hypocomplementemia anemia, elevated ESR and CRP levels, elevated D-dimers levels	This report describes a case of very-late-onset SLE with serositis as the predominant clinical feature, highlighting the need to consider SLE in the differential diagnosis of rapidly accumulating pleural effusion in elderly patients.
Hasegawa et al., 2023 1 case [7]	87 y/o ♂	anorexia, decreased food intake, bilateral pleural effusion, ascites, painful aphthous ulcers	positive tests for ANA hypocomplementemia leukopenia, thrombocytopenia, proteinuria	Diagnosing late-onset SLE in the elderly is challenging due to the prevalence of comorbidities and the need to distinguish it from other conditions. In some cases, conditions like pneumonia or heart failure may not fully explain the symptoms, leading to the eventual diagnosis of late-onset SLE through thorough testing and examinations.
Fujita et al., 2018 1 case [9]	70 y/o ♀	gait disturbance, cognitive dysfunction, muscle rigidity, bilateral pleural effusion, bilateral lower extremity edema	positive tests for ANA, anti-dsDNA Ab, anti-La/SS-B Ab hypocomplementemia lymphocytopenia, anemia, thrombocytopenia, elevated ESR and ferritin levels	Differentiating causes of cognitive impairment in elderly patients is challenging, with neurodegenerative diseases like Alzheimer’s being common considerations. Neuropsychiatric SLE should also be included in the differential diagnosis. Prompt and appropriate immunosuppressive therapy can potentially reverse cognitive dysfunction.
Kim et al., 2024 1 case [13]	81 y/o ♀	photosensitive lesions in sun-exposed sites	positive tests for ANA, anti-Ro/SS-A Ab	This case illustrates the development of SCLE after starting osimertinib for non-small-cell lung cancer. Prompt recognition and management of cutaneous side effects are crucial to maintaining targeted cancer therapy.
Mrabet et al., 2024 1 case [14]	82 y/o ♂	fatigue, dyspnea, abdominal pain, hepatosplenomegaly, pericardial effusion	positive tests for ANA, anti-nucleosome Ab, anti-histone Ab hypocomplementemia anemia, acute kidney injury, proteinuria, hematuria, hepatic cholestasis	Late-onset SLE often presents insidiously with atypical features, leading to delayed diagnosis, as seen in this case, who had abdominal pain and dyspnea without typical skin or joint signs. Moreover, this case demonstrates that familial late-onset SLE can occur in elderly men, presenting with a relatively mild form that does not necessitate intensive immunosuppressive therapy.
Tsuji et al., 2024 1 case [15]	75 y/o ♂	pleural effusion, pericardial effusion, gait ataxia, hand dexterity impairments, arthralgias	positive tests for ANA, anti-Ro/SS-A Ab, RF lymphocytopenia, anemia, elevated CRP levels	This case emphasizes that late-onset SLE can present with diverse symptoms, including unilateral pleural effusion and neurological issues, and should be considered when the cause of pleural effusion is unclear.
Rodriguez- Perez, 2024 1 case [16]	90 y/o ♀	recurrent unilateral pleural effusion, pericardial effusion, arthralgias, bilateral lower extremity edema	positive tests for ANA, p-ANCA anemia, marked proteinuria	This case emphasizes the significance of recognizing autoimmune conditions such as SLE in elderly patients over 90 y/o, with the presentation of marked proteinuria representing a rare and atypical manifestation.
Kioi et al., 2023 1 case [17]	86 y/o ♂	persistent unilateral pleural effusion	positive tests for ANA, anti-dsDNA Ab, anti-cardiolipin immunoglobulin G Ab, anti-U1-RNP Ab, anti-La/SS-B Ab, anti-CCP Ab lymphocytopenia, elevated CRP levels	The growing number of long COVID cases raises concerns about undiagnosed autoimmune conditions, such as lupus. In patients with persistent pleural effusion or lymphocytopenia post-COVID-19, testing for SLE-specific autoantibodies, including anti-dsDNA and anti-Sm Ab, should be considered.
Chao et al., 2022 1 case [18]	82 y/o ♀	ascites, pleural effusion, spontaneous oral bleeding, multiple ecchymoses	positive tests for ANA, anti-Sm Ab hypocomplementemia normocytic anemia, thrombocytopenia, renal dysfunction, proteinuria, elevated CA-125 levels	Pseudo-pseudo Meigs’ syndrome should be considered as a key differential diagnosis in female patients presenting with ascites, pleural effusion, and elevated CA-125 levels. The initial evaluation should focus on ruling out pelvic tumors associated with Meigs’ or pseudo-Meigs’ syndrome. An SLE flare should be considered, even in elderly patients.
Helali et al., 2022 1 case [19]	75 y/o ♂	weakness, dyspnea, respiratory failure	positive tests for ANA, anti-dsDNA Ab anemia, thrombocytopenia, proteinuria	Non-infectious fulminant lupus pneumonitis is a rare, severe syndrome with high mortality, with only a few cases reported in the literature, particularly in young patients. It is often challenging to diagnose, especially as an initial SLE manifestation.
Xu et al., 2021 1 case [20]	77 y/o ♂	unilateral pleural effusion, shortness of breath over the past 3 years	positive tests for ANA, anti-dsDNA Ab, MPO-ANCA anemia, renal dysfunction, proteinuria, microscopic hematuria	The overlap syndrome of SLE and antineutrophil cytoplasmic antibody-associated vasculitis is a rare condition. It is marked by the presence of serological markers and severe clinical features, such as rapidly progressive glomerulonephritis and pulmonary involvement, meeting the diagnostic criteria for both diseases. This syndrome predominantly affects women of childbearing age.
Kuroda et al., 2021 1 case [21]	78 y/o ♀	fatigue, diplopia, left sixth cranial nerve palsy, polyarthritis	positive tests for ANA, anti-dsDNA Ab, lupus anticoagulant hypocomplementemia leukopenia, anemia, elevated erythrocyte sedimentation rate (ESR) levels	This case highlights a challenging diagnostic scenario involving an elderly woman with autoimmune hemolytic anemia and isolated sixth cranial nerve palsy. The advanced age, low ANA titers, and atypical presentation made the diagnosis of SLE particularly complex.
Constantinescu et al., 2021 2 cases [22]	81 y/o ♀	vesperal fever, weight loss, arthralgias, myalgias, unilateral pleural effusion, pericardial fluid, atrophic erythematous squamous plaques	positive tests for ANA, anti-dsDNA Ab, anti-Ro/SS-A Ab leukopenia with lymphocytopenia, normocytic anemia	Skin lesions are less commonly reported in late-onset lupus, with malar rash being particularly rare in this group. However, the two cases discussed showed chronic and subacute cutaneous lupus lesions, later confirmed by skin biopsy.
	72 y/o ♀	weight loss, recurring fever, arthralgias, rounded erythematous squamous plaques	positive tests for ANA, anti-dsDNA Ab, RF leukopenia with lymphocytopenia, anemia	
Bao et al., 2021 1 case [23]	84 y/o ♂	bilateral peripheral edema	positive tests for ANA, anti-dsDNA Ab hypocomplementemia renal dysfunction, hypoalbuminemia, proteinuria, microscopic hematuria, pyuria	This case highlights a rare presentation of lupus nephritis as a paraneoplastic manifestation of mantle cell lymphoma. The rapid remission of renal and immune symptoms following chemotherapy supports the secondary nature of the lupus nephritis.
Tay et al., 2020 1 case [24]	71 y/o ♀	fever, alopecia, hepatomegaly, multiple intra-abdominal and intra-thoracic lymphadenopathies	positive tests for ANA, anti-dsDNA Ab leukopenia, anemia, renal dysfunction, microscopic hematuria, proteinuria	This case highlights the established connection between rheumatological disorders and cancers, as evidenced by unusual clinical features such as pronounced hepatomegaly and extensive lymphadenopathy, suggesting the presence of an underlying malignancy. Angioimmunoblastic T-cell lymphoma is a rare subtype of non-Hodgkin lymphoma. Its diagnosis is challenging due to subtle histological features.
Rezazadegan et al., 2020 1 case [25]	78 y/o ♀	papulosquamous itching skin lesions, weight loss, heartburn, arthralgias, myalgias	positive tests for ANA, anti-Ro/SS-A Ab hypocomplementemia anemia, biopsy of gastric mass showing gastric adenocarcinoma	Rheumatic diseases, like dermato-myositis, have been well established as paraneoplastic syndromes. More recently, growing evidence has linked SCLE to various malignancies, primarily solid tumors. These cancers may arise before, simultaneously with, or after the onset of SCLE.
Bonemetti et al., 2020 1 case [26]	85 y/o ♀	hemodynamic instability, diffuse dyscrasia edemas, peripheral cyanosis, particularly on her fingers, pleural effusion	positive tests for ANA, anti-Ku Ab, ANCA leukocytosis with lymphocytopenia, thrombocytopenia, renal dysfunction, elevated CRP and ferritin levels	This is the first documented case in the literature of SLE with vasculitis triggered by COVID-19 infection.
de Montjoye et al., 2020 1 case [27]	81 y/o ♀	impaired spatial and time orientation, dyspnea, weight loss, fever, arthritis, facial rash	positive tests for ANA, anti-dsDNA Ab, anti-Ro/SS-A Ab anemia, hypoalbuminemia, proteinuria, elevated CRP levels	Late-onset SLE is an uncommon but significant cause of reversible functional and cognitive decline in elderly patients. Its presentation in this age group is often atypical, making the diagnosis challenging.
Kalenchic et al., 2019 1 case [28]	75 y/o ♀	dyspnea, low-grade fever, arthritis, pericardial effusion, pleural effusion	positive tests for ANA, anti-dsDNA Ab, anti-nucleosome Ab lymphocytopenia, anemia, elevated ESR and CRP levels	This case illustrates that late-onset SLE can be a potential cause of pleural fluid accumulation in elderly patients.
D’Andréa et al., 2018 1 case [29]	82 y/o ♀	anorexia, weight loss, fatigue, arthralgia, unilateral pleural effusion	positive tests for ANA, anti-nucleosome Ab leukopenia with eosinophilia, anemia, elevated CRP levels, pleural effusion cytological examination showing Hargraves cells	In elderly patients, eosinophilic pleural effusion is most often linked to malignancies and infections. In rare instances, pleural eosinophilia may be associated with connective tissue diseases. When accompanied by chronic joint pain and hematologic abnormalities, SLE should be considered in the differential diagnosis.
Jatwani et al., 2018 1 case [30]	81 y/o ♂	shortness of breath, pericardial effusion, bilateral pleural effusion, bilateral lower extremity edema	positive tests for ANA, anti-dsDNA Ab, anti-beta-2 glycoprotein IgA, anti-CCP hypocomplementemia anemia, thrombocytopenia	Bronchiolitis obliterans organizing pneumonia (BOOP) is a rare manifestation of connective tissue disorders like SLE. In this case, BOOP was diagnosed 8 years before SLE. The high relapse rate, despite adequate steroid therapy, suggests a causal link to underlying SLE and indicates BOOP as the initial presentation.
Arai et al., 2018 1 case [31]	74 y/o ♂	fever, dyspnea, pericardial effusion, pleural effusion, arthritis	positive tests for ANA, anti-dsDNA Ab hypergammaglobulinemia, hypocomplementemia renal dysfunction	This report highlights a unique case of comorbid autoimmune diseases, featuring the coexistence of IgG4-related disease (IgG4-RD) and SLE. The clinical course demonstrated a transition from ANA-positive IgG4-RD to SLE after the surgical removal of gastric cancer.
Shirai et al., 2017 2 cases [32]	82 y/o ♂	bilateral polyarthritis	positive tests for ANA, anti-dsDNA Ab lymphocytopenia, mild thrombocytopenia, renal dysfunction, proteinuria	These two cases demonstrated that very-late-onset male SLE can present with atypical features, such as the absence of characteristic skin lesions, severe cytopenia, hypocomplementemia, anti-SS-A, anti-RNP, or anti-Sm antibodies, which complicate the diagnosis of SLE.
	83 y/o ♂	fatigue, dyspnea, bilateral pleural effusion, bilateral lower extremity edema	positive tests for ANA, anti-dsDNA Ab lymphocytopenia, hypoalbuminemia, elevated ESR and CRP levels	
Boddu et al., 2016 1 case [33]	73 y/o ♀	painful blistering skin rash, oral ulcers	positive tests for ANA, anti-RNP Ab, anti-Sm Ab hypocomplementemia anemia, thrombocytopenia, hypoalbuminemia, proteinuria	This case highlights the challenges physicians face in diagnosing among the numerous immunobullous dermatoses. Bullous SLE must be especially distinguished from pemphigoid and epidermolysis bullosa acquisita.
Kamiya et al., 2016 1 case [34]	83 y/o ♀	bilateral pleural effusion	positive tests for ANA, anti-dsDNA Ab, anti-RNP Ab hypocomplementemia proteinuria, elevated CRP levels, elevated CA-125 levels	Some studies have suggested a potential link between anti-RNP antibodies and lupus serositis. These findings indicate that lupus serositis should be considered in patients with anti-RNP-positive interstitial pneumonia.
Hammami et al., 2014 1 case [35]	77 y/o ♀	massive ascites, unilateral exudative pleuritis 4 years prior to presentation	positive tests for ANA, anti-dsDNA Ab, anti-Sm Ab hypocomplementemia leukopenia with lymphocytopenia, anemia, hypoalbuminemia	Massive ascites is a rare presentation of SLE. Lupus peritonitis may mimic acute abdominal conditions or present chronically as persistent painless ascites, chronic pancreatitis, or mild abdominal pain.
Agguire et al., 2014 1 case [36]	72 y/o ♂	right-sided chest pain, dyspnea, erythematous lesions on the sun-exposed areas, oral ulcers, bilateral lower and upper extremity edema, massive unilateral pleural effusion	positive tests for ANA, anti-dsDNA Ab, anti-RNP Ab hypocomplementemia leukopenia with lymphocytopenia, elevated CRP levels	Diagnosing late-onset SLE in elderly patients is challenging due to its nonspecific presentation and broad differential diagnoses. While elderly-onset SLE typically shows lower rates of anti-dsDNA, anti-RNP Ab, and hypocomplementemia, this case demonstrated elevated levels of these markers alongside complement consumption.
Chebbi et al., 2014 1 case [37]	82 y/o ♀	anorexia, weight loss, fatigue, arthralgia, unilateral pleural effusion	positive tests for ANA, anti-nucleosome Ab leukopenia with eosinophilia, anemia, elevated CRP levels, pleural effusion cytological examination showing Hargraves cells	In elderly patients, eosinophilic pleural effusion is most often linked to malignancies and infections. In rare instances, pleural eosinophilia may be associated with connective tissue diseases. When accompanied by chronic joint pain and hematologic abnormalities, SLE should be considered in the differential diagnosis.
Iyoda et al., 2008 1 case [38]	86 y/o ♂	general malaise	positive tests for ANA, lupus anticoagulant anemia, thrombocytopenia, elevated activated partial thromboplastin time, hypofibrinogenemia, elevated fibrin degradation products, elevated D-dimers, renal dysfunction, proteinuria, elevated CRP levels	The presence of disseminated intravascular coagulation (DIC) without an identifiable cause should raise suspicion for SLE, even in elderly patients. There have been only a few reports of DIC as the initial manifestation of SLE.
Ito et al., 2002 1 case [39]	77 y/o ♀	massive ascites, bilateral pleural effusion, pericardial effusion, bilateral lower extremity edema	positive tests for ANA, anti-dsDNA Ab hypocomplementemia leukopenia, anemia, renal dysfunction, hypoalbuminemia, proteinuria, microscopic hematuria	Chronic lupus peritonitis in elderly patients may show limited response to glucocorticoid therapy due to persistent peritoneal inflammation and impaired vascular function.
Aharon et al., 1994 2 cases [40]	70 y/o ♂	pleural effusion, generalized maculopapular rash 3 years prior to presentation	positive tests for ANA, anti-dsDNA Ab, anti-Sm Ab hypocomplementemia pancytopenia, renal dysfunction, proteinuria	SLE should be considered in elderly patients presenting with multiorgan autoimmune disease. For elderly patients where other immunosuppressive therapies are contraindicated, low-dose monthly intravenous immunoglobulin may be a treatment option.
	82 y/o ♀	anorexia, weakness, nodular purplish generalized skin rash, pericardial effusion, recurrent pleural effusion and recurrent chondritis over a period of 9 years prior to presentation	hypocomplementemia pancytopenia, renal dysfunction, elevated ESR levels	

♀, female; ♂, male; ANA, anti-nuclear antibody; anti-Ro/SS-A Ab, anti-Ro/Sjögren’s-syndrome-related antigen A antibodies; SCLE, subacute cutaneous lupus erythematosus; Ab, antibodies; SLE, systemic lupus erythematosus; RF, rheumatoid factor; p-ANCA, perinuclear anti-neutrophil cytoplasmic Ab; anti-dsDNA Ab, anti-double-stranded DNA antibodies; anti-U1-RNP Ab, anti-U1-ribonuclear protein antibodies; anti-La/SS-B Ab, anti-LA/Sjögren’s-syndrome-related antigen B antibodies; anti-CCP, anti-cyclic citrullinated peptide; anti-Sm, anti-Smith; CA-125, carbohydrate antigen 125; MPO-ANCA, amyeloperoxidase–anti-neutrophil cytoplasmic antibodies; ESR, erythrocyte sedimentation rate; AIHA, autoimmune hemolytic anemia; CRP, C-reactive protein; BOOP, Bronchiolitis obliterans organizing pneumonia; IgG4-RD, IgG4-related disease; DIC, disseminated intravascular coagulation.
